# The Vessels That Encapsulate Tumor Clusters (VETC) Pattern Is a Poor Prognosis Factor in Patients with Hepatocellular Carcinoma: An Analysis of Microvessel Density

**DOI:** 10.3390/cancers14215428

**Published:** 2022-11-03

**Authors:** Chun-Wei Huang, Sey-En Lin, Song-Fong Huang, Ming-Chin Yu, Jui-Hsiang Tang, Chi-Neu Tsai, Heng-Yuan Hsu

**Affiliations:** 1 Department of Surgery, New Taipei Municipal Tucheng Hospital, New Taipei City 23652, Taiwan; 2 Graduate Institute of Business Administration, College of Management, Fu Jen Catholic University, New Taipei City 242062, Taiwan; 3 Department of Pathology, New Taipei Municipal Tucheng Hospital, New Taipei City 23652, Taiwan; 4 College of Medicine, Chang Gung University, Taoyuan City 33305, Taiwan; 5 Division of Gastroenterology and Hepatology, Department of Internal Medicine, New Taipei Municipal Tucheng Hospital, New Taipei City 23652, Taiwan; 6 Graduate Institute of Clinical Medical Sciences, Chang Gung University, Guishan District, Taoyuan City 33302, Taiwan

**Keywords:** vessels that encapsulate tumor clusters (VETC), microvessel density (MVD), hepatocellular carcinoma (HCC)

## Abstract

**Simple Summary:**

Hepatocellular carcinoma has the characteristics of angiogenesis and neovascularization, which are the main steps in tumor growth and metastasis. The Vessels that encapsulate tumor clusters (VETC) pattern is a unique vascular structure in hepatocellular carcinoma. Microscopically, each tumor cluster is encapsulated by sinusoid-like vessels that form cobweb-like networks. Here, we retrospectively investigated the clinical–pathological features of hepatocellular carcinoma patients with or without the VETC pattern. We found that the VETC pattern is an independent factor of poor prognosis for long-term oncological outcomes. Meanwhile, higher intra-tumoral microvessel density was significantly associated with the VETC pattern. Further studies are needed to validate our findings.

**Abstract:**

The outcomes of patients with hepatocellular carcinoma (HCC) are unsatisfactory because of its high recurrence rate. The Vessels that encapsulate tumor clusters (VETC) pattern is a unique vascular structure. In this study, we investigated the clinical–pathological features of HCC patients with the VETC pattern. We retrospectively reviewed patients with HCC who underwent curative hepatectomy at Chang Gung Memorial Hospital between 2007 and 2013. The form of the VETC pattern was established using an anti-CD31 stain. The results were classified into positive (VETC^+^) and negative (VETC^−^) patterns. We investigated and compared demographic data between these two groups. Overall, 174 patients were classified into either the VETC^+^ or VETC^−^ groups. The median followed-up period was 80.5 months. There were significant differences in the number of hepatitis B carriers, the occurrence of vascular invasion, tumor size, TNM staging, microvessel density, and recurrence (all *p* < 0.05). Regarding the prediction of disease-free survival, after COX regression multivariate analysis, VETC^+^ remained independently associated with recurrent episodes (*p* = 0.003). The intra-tumoral microvessel density, demonstrated by CD-31, was the only clinical–pathological feature independently associated with VETC^+^. Our study demonstrated that the VETC pattern is an independent factor of poor prognosis for DFS. Higher intra-tumoral microvessel density was significantly associated with the VETC pattern. Further studies are needed to validate our findings.

## 1. Introduction

Hepatocellular carcinoma (HCC) is the fifth most common malignant disease and the second leading cause of cancer deaths worldwide [1]. Currently, outcomes for cancer-curative hepatectomy are unsatisfactory because of delayed diagnoses and high rates of recurrence [2]. Several cohort analyses have shown that the presence of microscopic features in surgical specimens, such as microvascular invasion (MVI), is related to early recurrence within 2 years after resection—whereas late recurrence after 2 years following resection has been linked to de novo malignancies that usually arise from chronically injured liver tissues [3,4]. Other pathologic variables have also been analyzed and correlated to tumor behaviors, such as encapsulation, intratumor steatosis, and tumor-infiltrating cells [5,6,7]. The tumor heterogeneity in HCC indicates that pathological subtypes could be helpful in the subclassification of HCC in addition to tumor staging, where glutamine synthase (GS), glypican-3 (GPC-3), heat shock protein 70 (Hsp70), and enhancer of zeste homologue 2 (EZH2) are the common markers for HCC diagnosis [8]. Biliary/stem cell markers and Wnt/β-catenin signaling have been used for the three-group classification of HCC [9]. Furthermore, microvessel density (MVD) is related to angiogenesis in terms of chemotaxis, which implies that the vascular pattern in tumors could reflect tumor aggressiveness [10,11].

Angiogenesis is important for both tumor growth and metastases in HCC, and this can be identified using the intra-tumoral microvessel density (MVD) [11,12]. Vessels that encapsulate tumor clusters (VETC) are another specific tumor vascular pattern that has been reported recently [13,14,15]. Microscopically, each tumor cluster is encapsulated by sinusoid-like vessels that form cobweb-like networks [16]. Knockdown of angiopoietin-2 (Ang2) could reduce the vascular pattern and decrease in vivo metastasis [13]. Fang and Xu et al. reported that the VETC pattern in patients with HCC is a predictor of beneficial effects in sorafenib treatment, and that this may be related to its antagonistic effect on Ang2 [14]. However, the mechanism of action of the VETC pattern has not been adequately studied.

Cluster of differentiation 31 (CD31) is an integral membrane glycoprotein that is expressed on endothelial cells in both normal tissues and tumors. Intra-tumoral MVD can be quantified based on CD31-positive endothelial cells in tumor samples and is often used as a surrogate for tumoral angiogenic activity. High intra-tumoral MVD may be linked to angiogenesis and aggressive tumor behavior [11].

In this study, we investigated the demographic distribution of HCC patients with and without the VETC pattern (VETC^+^/VETC^−^ HCC) and analyzed whether the VETC pattern could be a predictor of long-term outcomes in patients with HCC.

## 2. Materials and Methods

### 2.1. Study Population

This is a retrospective cohort study with 200 HCC patients who underwent curative partial hepatectomies between 2007 and 2013. Tumor staging was based on the 8th edition of the AJCC TNM staging system for HCC [17]. The multi-modality liver cancer team—composed of surgeons, hepatologists, radiologists, and pathologists—committed to twice-weekly meetings for treatment evaluation. In this study, the included patients’ ages ranged from 20 to 85 years and the exclusion criteria were unresectable disease, recurrent cancer, concurrent double cancer, and the presence of distant metastases at presentation. Major hepatectomies were defined as more than three segments requiring resection. The presence of complications was recorded when a higher than grade III Clavien–Dindo classification occurred, meaning that intervention or organ dysfunction occurred.

Patients with an undetermined VETC pattern or missing data were also excluded from this study (*n* = 26). Of 174 patients, the clinical demographic data and pathological variables including cirrhosis, vascular invasion, satellite lesions, and tumor grading were collected. Patients were divided into two groups by an independent pathologist, Dr. Lin, according to the presence of tumor clusters and vascular patterns using anti-CD31 staining (VETC^+^ group vs. VETC^−^ group). All patients underwent blood tests and triphasic CT scans of the liver 1 month after liver resection. Serial biochemical tests and imaging studies were performed and recorded every 3 months in the first 2 years and every 4–6 months thereafter. When recurrence—as indicated by triphasic CT—occurred, treatment was started based on the suggestions of the liver cancer team. Follow-up ended on 30 March 2019.

### 2.2. Pathological Analysis and Immunohistochemistry

The liver’s size, weight, and appearance were recorded. Corresponding formalin-fixed, paraffin-embedded liver specimens were then cut into 4 μm sections for histological staining. Hematoxylin and eosin (H&E) staining is the gold standard for medical diagnosis, while the detection of endothelial cells was based on CD31 staining. The microvessel density (MVD) was quantified as the number of CD31-positive vessels per millimeter square (vessels/mm^2^). The MVD of the tumor and non-tumor parts were calculated three times for each specimen and the average was recorded as the value of the MVD. CD31 staining was also used to examine the VETC pattern. A positive VETC pattern (VETC^+^) was defined as the presence of tumor clusters encapsulated by sinusoid-like vessels that formed cobweb-like networks, while those without any VETC pattern were identified as having a negative VETC pattern (VETC^−^). Encapsulation, satellite lesions, and vascular invasion were confirmed using both gross inspection and microscopy. Liver cirrhosis was defined using the Ishak fibrosis score.

### 2.3. Statistical Analysis

Pearson’s χ2 test was used to compare categorical variables. The Student’s *t* test was used to analyze continuous variables. The disease-free survival analysis was estimated for the VETC^+^ and VETC^−^ groups using the Kaplan–Meier method and the log-rank test. All significant factors in the univariate analysis were included in the COX regression multivariate analysis. Results from the multivariate analysis were demonstrated as hazard ratios (HR) and 95% confidence intervals (CI). A receiver operating characteristic (ROC) curve was developed to determine sensitivity and specificity. The optimal cut-off points were determined using Youden index estimation. All calculations were performed in SPSS statistics version 21.0 (IBM Corp., Somers, NY, USA). Two-tailed *p*-values < 0.05 were considered statistically significant.

## 3. Results

### 3.1. Patient Demographics and Pathological Variables

Of the 174 patients, 53 (30.5%) patients were in the VETC^+^ group and 121 (69.5%) patients were in the VETC^−^ group. The median follow-up duration was 80.5 months. The mean age of the study cohort was 60.7 ± 11.4 years and 125 patients (71.8%) were male. Hepatitis B and C (HBV and HVC) carriers included 85 (48.9%) and 89 (51.1%) individuals, respectively. The mean ICG-R15 was 10.4 ± 9.2% and the mean serum α-fetoprotein level was 2903.9 ± 32,517.2 ng/mL. Regarding pathological variables, 72 patients (41.4%) had liver cirrhosis according to their Ishak fibrosis score. Furthermore, 99.4% of the patients had Child–Pugh A, except for one patient with Child–Pugh B (0.6%). Additionally, 154 (89.1%) patients had encapsulated HCC and 22 patients (12.6%) had satellite lesions. Furthermore, 39 (22.4%) patients had macro or microvascular invasion. The mean tumor size was 3.8 ± 2.4 cm. The patients’ demographic data are summarized in Table 1.

### 3.2. Comparison of the VETC^+^ and VETC^−^ Groups

**Figure 1:**
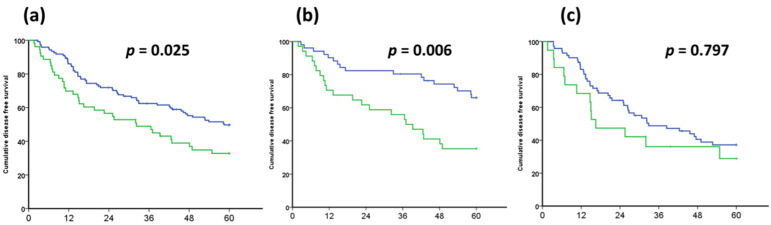
Kaplan–Meier survival curves of the VETC^+^ and VETC^−^ groups in patients with HCC who underwent partial hepatectomy. Different long-term outcomes of patients with HCC, stratified according to the presence or absence of the VETC pattern. The Kaplan–Meier analysis showed the disease-free survival in (**a**) the entire cohort, (**b**) the HBV group, and (**c**) the HCV group, from left to right. VETC, Vessels that Encapsulate Tumor Clusters; VETC^+^, positive VETC pattern; VETC^−^, negative VETC pattern; HCC, hepatocellular carcinoma; HBV, hepatitis B virus; HCV, hepatitis C virus; VETC^+^ group, green line; VETC^−^ group, blue line.

The clinical features and survival outcomes of HCC were distinct according to the presence or absence of the VETC pattern. There were significantly more HBV carriers in the VETC^+^ group than in the VETC^−^ group (64.2% vs. 42.1%; *p* = 0.008). Hepatocellular carcinoma in the VETC^+^ group had higher levels of vascular invasion, larger tumor size, and more advanced cancer stages (*p* = 0.002, *p* = 0.025, and *p* = 0.008, respectively), independently. The intra-tumoral MVD was significantly higher in the VETC^+^ group than in the VETC^−^ group (*p* < 0.001). Regarding the non-tumor part, there was no significant difference in MVD between the VETC^+^ and VETC^−^ groups (*p* = 0.096). Tumor recurrence, as a long-term oncological outcome, occurred significantly more often in patients in the VETC^+^ group (66.0% vs. 47.9%; *p* = 0.028). The detailed clinical and pathological data of the VETC^+^ and VETC^−^ groups are summarized in Table 1. Disease-free survival curves of the VETC^+^ and VETC^−^ groups were illustrated using the Kaplan–Meier method and the curves were compared using the log-rank test. As shown in Figure 1, compared to the VETC^−^ group (blue line), the VETC^+^ group (green line) had significantly poorer outcomes in all cohorts (*p* = 0.025; Figure 1a). In the subgroup analysis, there was a significant difference between the VETC^+^ and VETC^−^ groups in terms of HBV group but not HCV group (*p* = 0.006 and *p* = 0.797, respectively; Figure 1b and Figure 1c, respectively). Among patients with HCC who underwent partial hepatectomy, 53 (30.5%) patients in the VETC^+^ group (green line) had poorer outcomes compared to the 121 patients in the VETC^−^ group (blue line). In the subgroup analysis, disease-free survival—stratified according to the VETC pattern—was significantly different in the HBV group but not in the HCV group (*p* = 0.025, 0.006, and 0.797 from left to right).

### 3.3. Prediction of Recurrence by COX Regression Modeling

All significant factors in the univariate analysis were included in the multivariate analysis; these results are summarized in Table 2. The multivariate analysis showed that only ICG-R15 >15%, Edmondson–Steiner histology grade III and IV, the presence of liver cirrhosis, and VETC^+^ remained independently associated with tumor recurrence (*p* = 0.015, *p* = 0.015, *p* = 0.028, and *p* = 0.003, respectively). Patients in the VETC^+^ group had over two-fold higher odds of developing recurrent HCC compared to those in the VETC^−^ group (HR = 2.066; 95% CI, 1.280 to 3.337).

### 3.4. Prediction of VETC^+^ Hepatocellular Carcinoma

**Figure 2:**
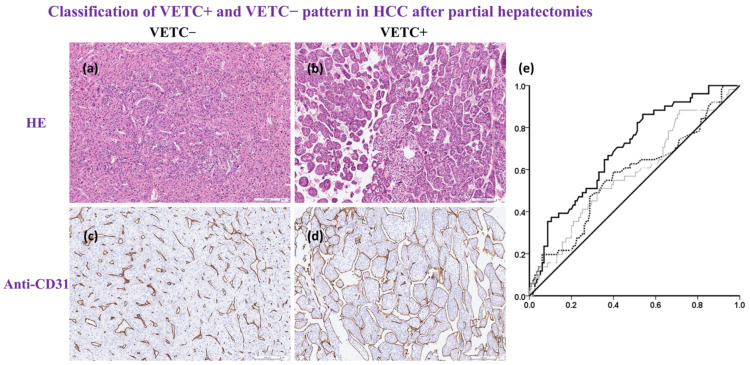
Histological photo based on Hematoxylin & Eosin stain or Anti-CD31; ROC curves for predicting VETC+. All surgical specimens were sent to and processed by a qualified pathologist. (**a**,**b**), H&E staining is the gold standard for medical diagnosis. Hypercellular density was noted in the VETC+ group, but the vascular structure was not clear on the H&E stain. (**c**,**d**), special anti-CD31 stain for the evaluation of intra-tumoral endothelial cells. The number of CD31-positive vessels/mm2 indicated intra-tumoral MVD. There were two distinct vascular patterns: (**c**), capillary-like vessels and disconnected blood vessels with small or no lumen indicated VETC−; (**d**) vessels that encapsulated tumor clusters and formed a cobweb-like pattern indicated VETC+; scale bar, 100 μm. (**e**) ROC curves for predicting VETC+ according to intra-tumoral MVD, the MVD of the normal part of the liver, and serum α-fetoprotein (black line, dotted line and grey line, respectively). Only intra-tumoral MVD independently predicted VETC+ (AUROC = 0.693; 95% CI, 0.613 to 0.780, *p* < 0.001). When the cut-off score was 40 vessels/mm2, the sensitivity and specificity of VETC+ was 0.863 and 0.461, respectively. Anti-CD31, cluster of differentiation 31 antibody; ROC curve, receiver operating characteristic curve; VETC+, positive VETC pattern; H&E stain, Hematoxylin & Eosin stain; MVD. Microvessel density; VETC−, negative VETC pattern, AFP, α-fetoprotein; Intra-tumoral MVD, black line; Normal part liver MVD, dotted line; Serum AFP, grey line.

Patients with VETC^+^ HCC had only slight hypercellular density with H&E staining (Figure 2a,b). The appearance of a cobweb-like network was identified using anti-CD31 staining (Figure 2c,d). All clinical and pathological factors were used to predict VETC^+^ HCC. The receiver operating characteristic (ROC) curve and the area under the ROC curve (AUROC) are shown in Figure 2e. Only intra-tumoral MVD identified using CD31 was shown to be a predictor of VETC^+^ HCC (AUROC = 0.693; 95% CI, 0.613 to 0.780, *p* < 0.001). The optimal cut-off value was determined with the Youden index and when the cut-off score of the intra-tumoral MVD was 40 vessels/mm^2^, the sensitivity and specificity for VETC^+^ HCC was 0.863 and 0.461, respectively. The MVD of the non-tumor parts of the liver and serum α-fetoprotein levels were not significantly associated with VETC^+^ HCC (for the MVD of the non-tumor parts of the liver, AUROC = 0.572, 95% CI, 0.475 to 0.669, *p* = 0.138; for serum α-fetoprotein, AUROC = 0.583, 95% CI, 0.487 to 0.678, *p* = 0.090).

## 4. Discussion

The results of our study indicate that VETC^+^ HCC shows more aggressive tumor behavior compared to VETC^−^ HCC and that the presence of the VETC pattern is independently associated with HCC recurrence. Compared to patients with VETC^−^ HCC, patients with VETC^+^ HCC had a higher probability of developing multiple liver recurrence and distant metastasis (32.4% vs. 22.5%), including locoregional recurrence, distant metastases, or de novo malignancies from chronic liver injury. Differences between VETC^+^ and VETC^−^ HCC were mostly present in terms of their pathological features. In our study, patients with VETC^+^ HCC had significantly larger tumors and higher levels of vascular invasion. Studies have indicated that VETC^+^ HCC has a short doubling time and tends to invade surrounding vessels. These pathological features result in advanced cancer stages at diagnosis; hence, patients with VETC^+^ HCC are expected to have a worse prognosis.

The high prevalence of HBV carriers in the VETC^+^ group is also important. In the subgroup analysis, it was the HBV and not the HCV group that had a significant difference in disease-free survival between the VETC^+^ and VETC^−^ groups (Figure 1a,b). A multi-institutional study that collected data from 541 cases showed that VETC^+^ is less common in Japanese populations (8.7%) but that it is present in 18.9% of the overall population [15].

Integrated hepatitis B virus X (HBx) genes from HBV tend to express viral gene products when insertional mutagenesis occurs. Integrated hepatitis B virus X (HBx) genes tend to express viral gene products. HBx proteins raise the mRNA levels of the epidermal growth factor receptor (EGFR) and modulate the vascular endothelial growth factor (VEGF), which ultimately induces carcinogenesis [18]. The Hepatitis B virus can also cause HCC in non-cirrhotic livers. Moreover, HBV-related HCC rapidly grows and is more likely to be diagnosed at younger ages compared to HCV [19]. This is compatible with our finding that indicates an independently higher prevalence of HBV carriers in the VETC^+^ group and that they have worse outcomes. Further prospective and well-designed studies are needed to support this conclusion.

Hepatocellular carcinoma is a typical hypervascular and solid lesion in the liver. As the tumor expands, the diffusion distance from the existing supplying vessels increases, leading to hypoxia. Angiogenesis is a result of hypoxic stress and can promote tumor proliferation, migration, and invasion—which ultimately leads to metastasis and tumor survival [10]. HBV-HCC with a rich blood supply has a growth advantage and can metastasize early on through the interaction that occurs between caveoli-1, VEGF, and MVD [20]. Previous reports have shown that Ang-2 is a key molecule for the development of the VETC pattern, and that it is also related to increased MVD and expression of VEGF in hypoxic conditions [21]. Furthermore, VETC has been associated with increased MVD and angiogenesis. Therefore, anti-angiogenesis therapy in HCC treatments has been developed for BCLC stage C and refractory TACE [22]. Sorafenib (Nexavar^®^) and Lenvatinib (Lenvima^®^), two multi-kinase inhibitors with potent anti-angiogenic capacity, have been used as first-line therapies for hepatocellular carcinoma in the past [23,24]. The VETC pattern used has also been shown to be a predictive biomarker for sorafenib treatment efficacy [13,14,15].

Quantifying angiogenesis in vivo is difficult, and most studies have focused on surgical specimens. However, intra-tumoral MVD has been shown to be an emerging and promising biomarker in several cancers. Previous studies in the literature and meta-analyses have also verified its role in HCC. Endothelial cells can be detected and merged using anti-CD31 or anti-CD34, and this is used to calculate MVD in vitro. In this study, intra-tumoral MVD was significantly different between the VTEC^+^ and VETC^−^ groups (*p* < 0.001). However, there was no difference in the MVD of the non-tumor part. Increasing MVD and angiogenesis are the main steps in the carcinogenesis of HCC. A positive VETC pattern, as a strong and useful biomarker, was recognized in the laboratory by a qualified pathologist. Although this difficult and repetitive work can be made easier by artificial intelligence in the future, in this study, it was performed by a human being. The ROC curve indicated that only intra-tumoral MVD was independently associated with and could predict VETC^+^. Another well-known serum biomarker for HCC is α-fetoprotein; however, although it is technically accessible, it does not play a role in predicting VETC^+^.

The limitations of the current study include its retrospective design and small number of patients. Selection bias was also inevitable and could have affected the statistical results. All the patients in our study were disease-free after curative hepatectomy. No adjuvant therapy has been shown to have any evident advantage in stage I to II patients. Furthermore, most previous studies have focused on the treatment benefits of sorafenib in patients with VETC^+^ HCC; however, our study demonstrates the distinct pathological features of VETC^+^ HCC and is the first study to use intra-tumoral MVD to predict VETC^+^ HCC.

## 5. Conclusions

In conclusion, our study demonstrates that VETC^+^ is a significant factor of poor prognosis in patients with HCC after partial hepatectomy (Table 3). A positive VETC pattern was also independently associated with recurrent HCC and poorer disease-free survival. Most of the significant differences between the VETC^+^ and VETC^−^ groups were in pathological variables including tumor size, vascular invasion, intra-tumoral MVD, and TNM stage. In the subgroup analysis, HBV and not HCV was significantly different between the VETC^+^ and VETC^−^ groups. Furthermore, intra-tumoral MVD was shown to be the only clinical–pathological factor that was suitable for predicting VETC^+^. More prospective trials are required to confirm our conclusions.

## Figures and Tables

**Table 1:** Demographic data and clinical correlations with VETC classification for HCC patients after partial hepatectomies.

Variables	All (*n* = 174)	VETC Positive (*n* = 53)	VETC Negative (*n* = 121)	*p* Value
Age (yr)	62.6 (53.9~68.9)	60.6 (52.0~68.0)	62.2 (54.5~69.3)	0.371
Sex (male)	125 (71.8)	39 (73.6)	86 (71.1)	0.735
Comorbidity (yes)	89 (51.1)	31 (58.5)	54 (44.6)	0.092
HBV positive	85 (48.9)	34 (64.2)	51 (42.1)	0.008
HCV positive	89 (51.1)	19 (35.8)	70 (57.9)	
ICG R15	8.3 (4.0~13.4)	7.7 (3.8~11.0)	8.4 (4.1~14.3)	0.178
Major hepatectomy	32 (18.4)	13 (24.5)	19 (15.7)	0.167
Anatomic resection (*n* = 149)	48 (32.2)	12 (28.6)	36 (33.6)	0.551
AST (IU/L)	38.0 (26.8~63.3)	37.0 (26.5~52.0)	39.0 (27.0~70.0)	0.497
ALT (IU/L)	40.5 (27.0~74.3)	37.0 (27.0~53.0)	43.0 (28.0~82.5)	0.300
ALP (IU/L)	74.0 (59.0~94.0)	72.0 (53.5~95.5)	76.0 (60.0~94.0)	0.783
BIL (mg/dl)	0.6 (0.5~0.8)	0.6 (0.5~0.8)	0.6 (0.4~0.8)	0.677
ALB (g/dl)	4.2 (3.9~4.5)	4.2 (3.9~4.5)	4.2 (3.9~4.5)	0.343
AFP (ng/mL)	11.7 (4.6~83.8)	26.8 (5.9~149.4)	10.5 (3.9~56.4)	0.280
AFP (>200 ng/mL)	29 (16.7)	10 (18.9)	19 (15.7)	0.606
Cirrhosis	72 (41.4)	31 (58.5)	71 (58.7)	0.982
OP time (min)	250 (202~323)	249 (215~319)	255 (195~325)	0.835
Blood loss (mL)	225 (100~537)	300 (100~800)	300 (100~500)	0.159
Encapsulation	155 (89.1)	49 (92.5)	106 (87.6)	0.435
Satellite lesion	22 (12.6)	8 (15.1)	14 (11.6)	0.520
Vascular invasion				
No	135 (77.6)	32 (60.4)	103 (85.1)	0.002 **
Microvascular	30 (17.2)	16 (30.2)	14 (11.6)	
Gross	9 (5.2)	5 (9.4)	4 (3.3)	
Tumor size >5 cm	29 (16.7)	13 (24.5)	16 (13.2)	0.066
Tumor size (cm)	3.2 (2.2~4.5)	3.7 (2.6~5.7)	3.0 (2.2~4.0)	0.025 *
Grade III, IV/II, I	67 (38.5)/107 (61.5)	20 (37.7)/33 (62.3)	47 (38.8)/74 (61.2)	0.890
AJCC 8 staging IA/IB/II	32 (18.4)/84(48.3)/58(33.3)	5 (9.4)/22 (41.5)/26 (49.1)	27 (22.3)/62 (51.2)/32 (26.4)	0.008 **
Microvessel density (anti-CD31 staining)				
Tumor	49.0 (27.0~75.3)	60.3 (42.8~94.0)	43 (22.2~69.3)	<0.001 ***
Non-tumor part	11.7 (7.0~21.0)	14.7 (7.1~21.7)	10.5 (7.0~21.0)	0.096
Recurrence (yes)	93 (53.4)	35 (66.0)	58 (47.9)	0.028 *

Continuous variables are expressed as median (interquartile range) while categorical variables are expressed as number (%); HBV: hepatitis B virus; HCV: hepatitis C virus; AST: aspartate aminotransferase; ALT: alanine aminotransferase; ALP: alkaline phosphatase; BIL: bilirubin; ALB: albumin; AFP: alpha-fetoprotein; AJCC 8 staging: the 8th edition of American Joint Committee on Cancer (AJCC) TNM staging system. * *p* < 0.05, ** *p* < 0.01, *** *p* < 0.001.

**Table 2:** Clinicopathologic data and VETC expression of 174 HCC patients in univariate and multivariate regression analysis.

Variable	Univariate Analysis			Multivariate Analysis		
	HR	95% CI	*p* Value	HR	95% CI	*p* Value
Age (yrs) >70 (20.1%) vs. ≤70 (79.9%)	1.767	1.109–2.818	0.017	1.535	0.925–2.548	0.097
Sex (M/F) F (28.2%) vs. M (71.8%)	0.735	0.459–1.177	0.199			
Comorbidity Yes (51.1%) vs. No (48.9%)	1.101	0.733–1.654	0.644			
Hepatectomy Major (18.4%) vs. Minor (81.6%)	1.567	0.670–1.919	0.638			
ICGR15 (15%) High (18.8%) vs. Low (81.2%)	1.885	1.164–3.052	0.010 *	2.056	1.151–3.673	0.015 *
Complication Yes (13.5%) vs. No (86.5%)	1.681	0.960–2.945	0.069			
OP time (300 min) More (32.8%) vs. less (67.2%)	1.233	0.809–1.881	0.330			
Blood Loss (500 mL) More (25.0%) vs. less (75.0%)	1.651	1.063–2.564	0.026	1.115	0.681–1.824	0.666
Tumor size (cm) >5.0 (16.7%) vs. ≤5.0 (83.3%)	1.833	1.105–3.040	0.019 *	1.510	0.821–2.780	0.185
Satellite lesions (%) Yes (12.6%) vs. no (87.4%)	1.354	0.753–2.433	0.311			
Vascular invasion (%) Gross (5.2%) vs. Micro (17.2%) vs. No (77.6%)	1.856	1.334–2.582	<0.001 ***	1.230	0.783–1.934	0.369
Grading I/II/III, IV (%) III, IV (38.5%) vs. I, II (63.5%)	1.606	1.068–2.417	0.023 *	1.724	1.111–2.674	0.015 *
Cirrhosis (%) Yes (41.4%) vs. no (58.6%)	1.656	1.071–2.558	0.023 *	1.704	1.058–2.747	0.028 *
Encapsulation Yes (89.1%) vs. no (10.9%)	1.069	0.569–2.006	0.836			
a-fetal protein; AFP (200 ng/mL) >200 (16.7%) vs. ≤200 (83.3%)	1.482	0.875–2.512	0.143			
AJCC 8th Stage ^a^ II/III (33.3%) vs. IB (48.3%) vs. IA (18.4%)	1.728	1.270–2.350	<0.001 ***	1.322	0.892–1.961	0.165
AST (IU/L) 2ULN >68 (21.8%) vs. ≤68 (78.2%)	1.921	1.226–3.010	0.004	0.815	0.375–1.771	0.605
ALT (IU/L) 2ULN >72 (25.9%) vs. ≤72 (74.1%)	1.898	1.236–2.915	0.003	1.819	0.915–3.617	0.088
ALP (IU/L) >100 (18.4%) vs. ≤100 (81.6%)	1.511	0.929–2458	0.096			
ALB (g/dl) >3.5 (94.8%) vs. ≤3.5 (5.2%)	0.505	0.220–1.158	0.107			
VETC Positive (30.5%) vs. Negative (69.5%)	1.714	1.126–2.609	0.012	2.066	1.280–3.337	0.003 *

* statistical significance (*p* < 0.05) and *** for *p* < 0.001; HR, hazard ratio; 95% CI, 95% confidence interval of hazard ratio. Disease-free survival was calculated by univariate and multivariate Cox regression analysis. ^a^: American Joint Committee on Cancer, 8th tumor staging.

**Table 3:** Summary of the presence of VETC in HCC after resection for disease-free survival.

Factor	HR (95% CI)		Estimated Median Survival (Months)
ICGR15	2.056 (1.151–3.673)	High	22.6
		Low	53.9
Histology grading	1.724 (1.111–2.674)	III, IV	32.9
		I, II	58.2
Cirrhosis	1.704 (1.058–2.747)	Yes	42.6
		No	Not met
VETC	2.066 (1.280–3.337)	Yes	42.6
		No	Not met

HR, hazard ratio; 95% CI, 95% confidence interval of hazard ratio.

## Data Availability

The data presented in this study are available on request from the corresponding author.
